# 100 generations of wealth equality after the Neolithic transitions

**DOI:** 10.1073/pnas.2400697122

**Published:** 2025-04-14

**Authors:** Tim Kerig, Enrico R. Crema, Jennifer Birch, Gary M. Feinman, Adam S. Green, Detlef Gronenborn, Dan Lawrence, Cameron A. Petrie, Paul Roscoe, Amy E. Thompson, Timothy A. Kohler

**Affiliations:** ^a^Cluster of Excellence Roots, Kiel University, Kiel 24118, Germany; ^b^Historisches Seminar, Ur- und Frühgeschichte, University of Leipzig, Leipzig 04109, Germany; ^c^Department of Archaeology, University of Cambridge, Cambridge CB2 3ER, United Kingdom; ^d^Department of Anthropology, University of Georgia, Athens, GA 30606; ^e^Neguanee Integrative Research Center, Field Museum of Natural History, Chicago, IL 60605; ^f^Department of Anthropology, University of Illinois-Chicago, Chicago, IL 60607; ^g^Department of Archaeology, University of York, King’s Manor, York YO1 7EP, United Kingdom; ^h^Department of Environment and Geography, University of York, King’s Manor, York YO1 7EP, United Kingdom; ^i^Leibniz-Zentrum für Archäologie, Mainz 55116, Germany; ^j^Department of Archaeology, Durham University, Durham DH1 4HW, United Kingdom; ^k^Department of Anthropology, University of Maine, Orono, ME 04469; ^l^Climate Change Institute, University of Maine, Orono, ME 04469; ^m^Department of Geography and the Environment, The University of Texas at Austin, Austin, TX 78712; ^n^Department of Anthropology, Washington State University, Pullman, WA 99164-4910; ^o^Santa Fe Institute, Santa Fe, NM 87501; ^p^Crow Canyon Archaeological Center, Cortez, CO 81321

**Keywords:** archaeology, neolithic, social inequality, comparative archaeology, economic archaeology

## Abstract

From Rousseau onward, scholars have identified the transition to sedentary agriculture as crucial to the history of wealth inequality. Here, using the GINI project’s global database on disparities in residential size, we examine the effects of important innovations in plant cultivation, animal husbandry, and traction on wealth inequality. Over a series of regional case studies, we find no evidence of major changes in residential disparity before or after these technological innovations became widespread, and where the effects of systemic change are recognizable, they are ambiguous. The introduction of horticulture/farming is accompanied by a slight general increase in inequality, while subsequent innovations tend to have a leveling effect. Although increasing productivity and surplus are critical to generating wealth inequality, nothing in our data suggests that rising productivity alone led to greater wealth inequality.

## Significance

Social inequality and productivity have never been greater than they are today, and there is likely a connection between the two. Focusing on 2,000 y before and after the transition to the new production mode that defined the Neolithic, we examined this relationship across a variety of spatiotemporal contexts. Are increasing inequalities correlated in time with increased food production considered to be the most important change in preindustrial economic history? Does the development of higher productivity and social inequality take place in the same way everywhere, or does it follow different pathways? We identify specific conditions of equality that were present at the beginning of humanity’s march toward today's heightened inequalities while emphasizing the fundamental indeterminacy of their development.

Did rising Neolithic productivity inevitably and directly lead to rising inequality (here defined as the differential accumulation of wealth)? Has innovation always been motivated by individual profit? Does a successful economic innovation necessarily lead to an unequal distribution of the newly generated surplus? We address these questions using disparities in residential size as a proxy for Neolithic wealth inequality. We examine the global pattern and six case studies (*SI Appendix*) from the 2,000 y after the respective regional transition from an economy based on hunting and gathering to one based on husbandry, horticulture, and/or agriculture. Across six case studies, we examine the advance of wealth inequality in the 2,000 y following the transition from a hunter-gatherer economy to one based on husbandry, horticulture, and/or agriculture. We analyze the temporal relation between the development of residential disparity and developments commonly associated with significant productivity increases.

A recently published study (1) identifies three ways of making use of such Neolithic surplus: it can be consumed directly; it can be exchanged to acquire goods; or it can be skimmed off by a few, thereby increasing social inequality. In such scenarios, Neolithic economies’ surpluses are seen as inevitably leading to more durable private property (1, 2). For the first two cases, however, this is not necessary: surpluses are divisible among equals. In the following, we examine and criticize this argument and add another scenario to the three mentioned above. We add the ethnographically and historically documented scenario of nonincreased production and consumption volumes even under rising labor productivity (yield per time worked) (3). At first sight, research on inequality seems polarized (4), although in reality the division is more ideological than logical (5). Politically conservative approaches tend to focus on the benefits of social inequality as a driver of history. Higher individual returns incentivizing higher risks while social inequality ultimately leads to societal stability. The argument holds that surplus production facilitates increased social scale, with inequality emerging as an institutional element to manage and coordinate the process (e.g., refs. 6, 7, cf. refs. 8, 9). In this perspective, wealth inequality enables and stabilizes larger, more complex, and functionally advantageous societies (10); the common interest (9) therefore outweighs demands for an equal distribution of wealth; and radical disruptions are required (2) to reset wealth distributions. A related position, also originating in conservative thought, sees increasing surplus and wealth distribution as a repetitive and cyclical process. Wealth accumulation by emerging elites during integrative phases stabilizes society, but societies eventually shift into a disintegrative phase, with wealth becoming reordered and possibly also destroyed (e.g., refs. 11, 12). Politically progressive positions, ultimately originating in revolutionary or participatory movements of the last two centuries, focus on surplus-induced inequalities as generating internal contradictions (e.g., ref. 13), asymmetrical participation (14), and/or social dysfunctions (15, 16) that can lead to social collapse.

Here, we take an alternative position, emphasizing the difference between necessary and sufficient conditions—between the potential for greater wealth inequalities and the realization of inequality. We suggest that whether one translates into the other depends on variations in the nature of human action, institutions, and governance (17, 18). We show that important qualitative leaps in productivity may have had no direct influence on the development of inequalities, thereby opening up the role of agency and the historically specific in human affairs.

By inequality, we mean the accumulation of wealth in certain parts of a society. By productivity, we refer to labor productivity (yield per time worked). Using the GINI database of residential disparities (19, 20)—differences in residential unit size within a settlement—we evaluated temporal patterns of Neolithic inequality over a period that saw three key economic innovations that should have increased surplus production significantly (21, 22): agriculture, animal husbandry, and traction. We conducted both a global comparison (nine regions, see Fig. 3) (*Materials and Methods*) and a regional analysis of six regions that offer the most complete and thus meaningful data (Section 1 and *SI Appendix*).

We calculated Gini indices for the settlements in our survey, i.e., measures of deviation of residential unit sizes in a settlement from a hypothetical equality, a proxy for inequality shown to be suitable for global use (21, 23, 24). Those Gini indices generally measure longer-term household investments and—with regard to the minimum required domestic economic area—are more strongly linked to the functional necessities of production (25) than, for example, hoard finds or graves. Area dimensions can also be easily compared and analyzed. For example, in comparison to, e.g., grave furnishings, residential units’ sizes depict to a lesser extent events of individual social drama, in which supposed or actual social inequality is sometimes demonstrated by grave furniture to an audience (26). Instead, residential units’ sizes are determined by actual minimum needs for the long-term functioning of households (5).

Although house sizes are undoubtedly affected by the number of people inhabiting a residence and by practices such as housing animals within residences. Such practices tend to be widespread within contemporaneous households and are thus controlled for by sampling archaeological residences within settlements.

Household size can also fluctuate due to generational changes and cycles of household formation, expansion, and contraction, with short-lived buildings reflecting these fluctuations more clearly than more permanent ones. Buildings that are more sensitive to size fluctuations will therefore result in higher Gini indices. Variation in intrasettlement economic strategies may also cause variation in building size and Gini indices. In these cases, the error is asymmetrical at best: in the absence of high Gini indices (as we will document), there is therefore little risk of distortion due to the short lifespan of certain buildings.

For its part, the buildings enforce practices with which this social inequality can be recognized or undermined (23). Of course, the availability or the scarcity of living space is not the same in all societies at all times. In special settlement locations, such as tells, bays, or hilltops, there is a shortage of space which might not be the case in open landscapes for example. The calculation of the Gini coefficient allows a comparison of the concentration of the values for household areas within and between settlements (27).

## 1. Results

**Fig. 1.**
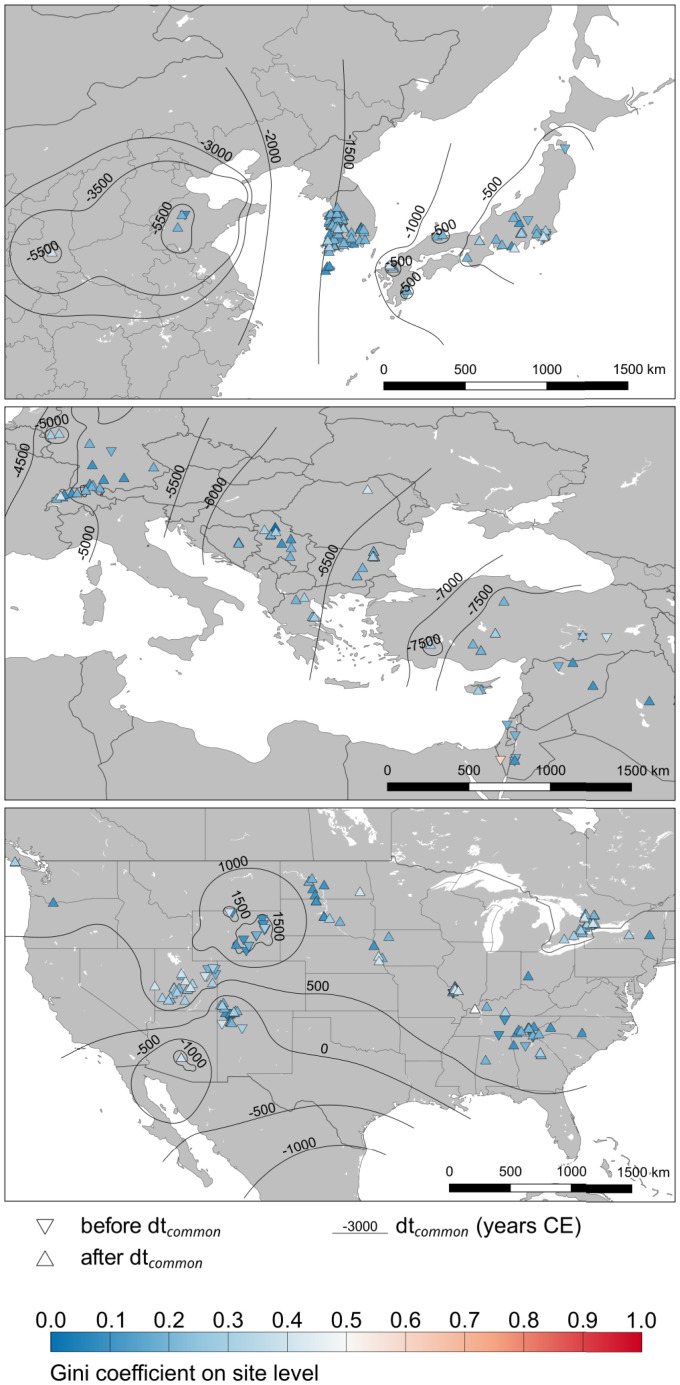
Neolithic transition inequality. Gini coefficients at site-level against the spread of the Neolithic. Shown are the 2,000 y before and after domesticated plants became commonly produced (dt common). Isolines of dt common are calculated from dt dates of the sites (method: inverse distance, modified, see *SI Appendix*).

In the following, the distribution of Gini coefficients computed from residential-unit size (20, 21, 27) was analyzed over the two millennia before and following the introduction of plant cultivation (Fig. 1). We used what we consider the most important production innovations as milestones in the development of global preindustrial production. For comparative purposes, the first occurrences by region were set to zero (dt = 0), following (28). We consider plant management/crop production (dt), animal management/herding (dt2), and animal traction (dt3) and observe the points in time at which they became widespread. The first regional occurrence [e.g., (Plant.cultivation…earliest); brackets here and in the following indicate columns in the GINI database] is to be understood as the date of the technological invention. The date when the innovation becomes common [(Plant.cultivation…common)] is when the innovation wrought complex systemic changes and is benchmarked as involving more than 50% of plants produced (29). These classifications of earliest and common are based on our data collectors’ expert knowledge and follow GINI project specifications (30). We analyze a global dataset and discuss those six meaningful regional case studies for which we could find data at least for the first 1,000 y after the respective time of common crop production (*SI Appendix*, Fig. S3).

### 1.1. Box Plots.

**Fig. 2.**
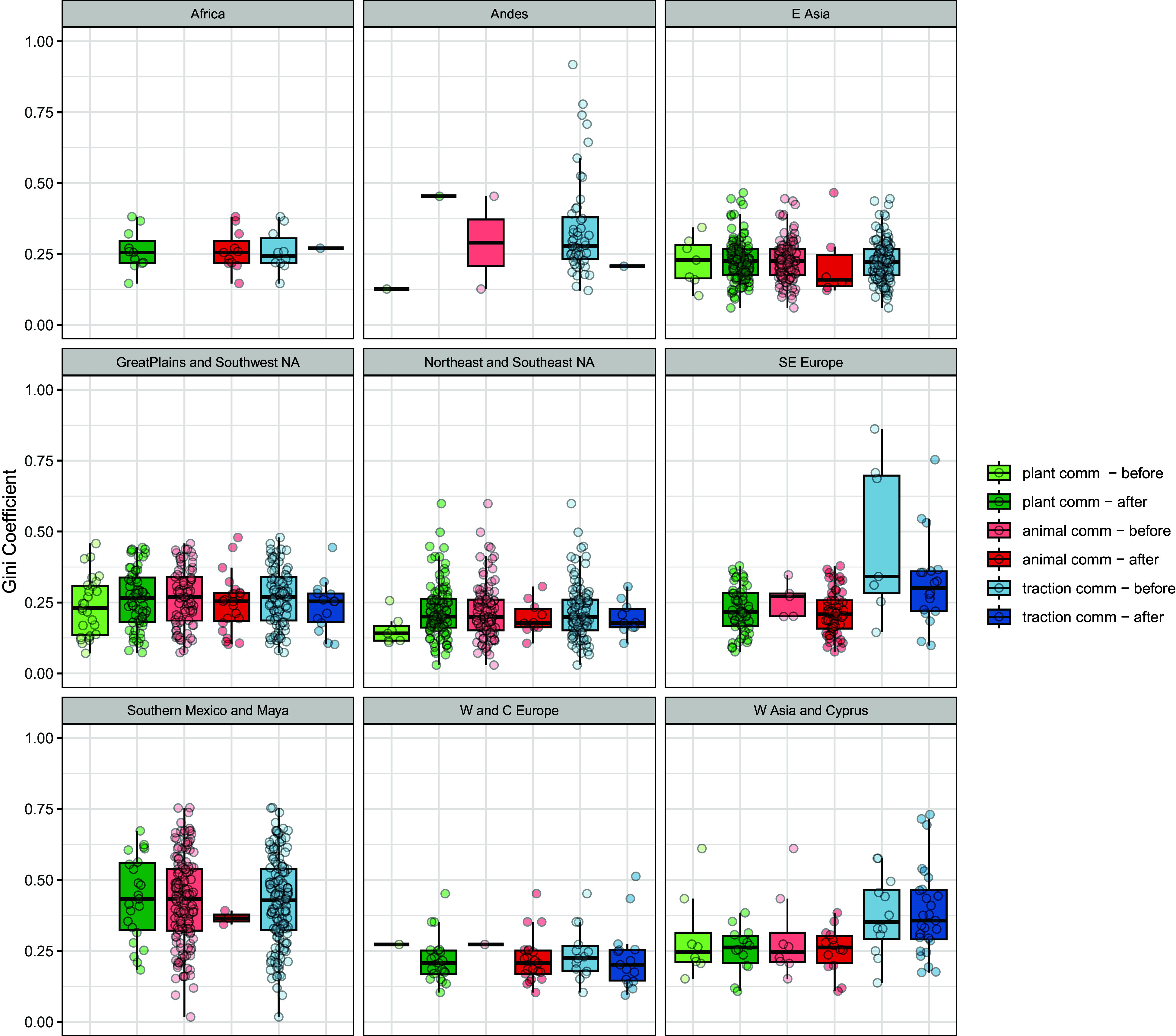
Neolithic transition inequality. Box plots of Gini coefficients at site-level by regions, before and after domesticated plants, animals, and traction became common.

Box plots for Gini coefficients by region, comparing 2,000 y before and after dt, dt2, and dt3 (Fig. 2) show generally similar distributions. The comparably moderate medians of Gini coefficients are mainly between 0.2 and 0.3 with slightly higher Gini coefficients in W Asia and Cyprus and, due to outliers, in SE Europe. In W Asia, dt and dt2 correspond, while moderately higher Gini coefficients are connected with dt3, an effect of long duration with weakly increasing Gini coefficients (*SI Appendix*, Figs. S3–S5).

### 1.2. Beta Regression.

**Fig. 3.**
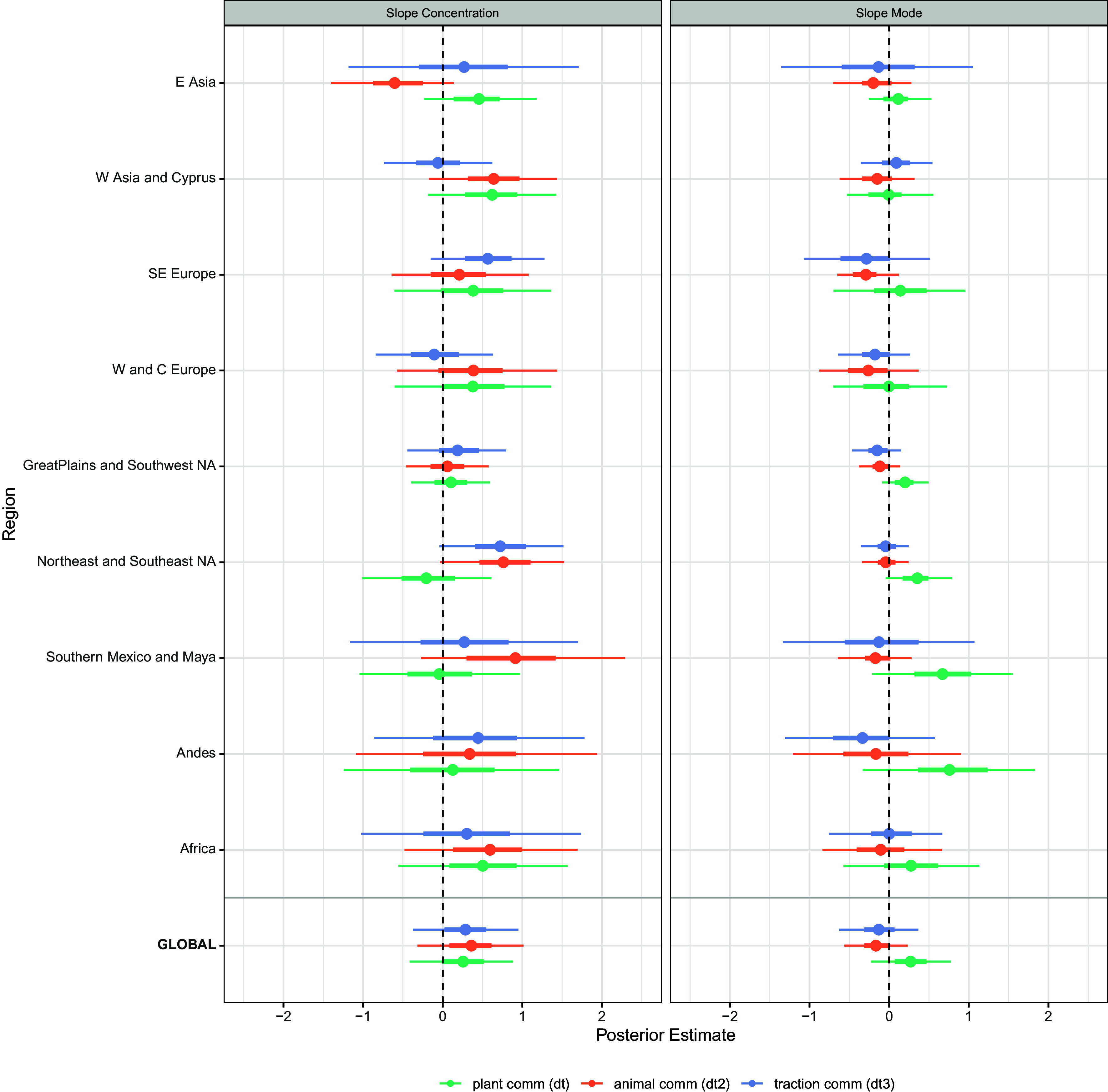
Neolithic transition inequality. Result of the beta regression: location and dispersion parameters of posterior estimates (additional information on case studies in *SI Appendix*).

The hierarchical beta regression analyses (*Materials and Methods*) examined both changes in the central tendency (mode parameter, in the following Slope Mode) and dispersion (concentration parameter, in the following Slope Concentration) of the Gini coefficients 2,000 y before and after dt, dt2, and dt3. Fig. 3 shows 90 and 50% highest posterior density intervals (HPDI), or credible intervals, of the slope parameters. Positive values for Slope Mode indicate that the central tendency of the Gini coefficients was higher after the particular economic change became common, while positive values for Slope Concentration suggest a decreased variance (i.e., increased concentration) in the residential disparities. The 90% HPDI includes 0 in all cases, suggesting that we do not have sufficient evidence to argue for a robust signal of change in Gini coefficients after dt, dt2, and dt3. An overlap between 0 and the 90% HPDI can be both the result of an absence of evidence (e.g., wider posterior ranges resulting from small sample sizes, e.g. *Andes*), or evidence of absence (e.g., narrow posteriors ranges close to zero, e.g., slope parameter for the mode in *W Asia and Cyprus*).

Notwithstanding high levels of uncertainty, our analyses do provide some tentative findings. Slope Mode generally shows values close to zero even when sample sizes are comparatively large for all dt, dt2, and dt3, although both global and regional signals for dt (e.g., from all regions in the Americas) seem to point toward an increase in the Gini coefficient, with the 50% HPDI above 0. With few exceptions, dt2 and dt3 generally show negative Slope Modes, indicating that the Gini coefficient decreases its modal value after dt2 and dt3. Yet in both cases, the 90% HPDI of the slope includes zero.

Results for the concentration parameter, Slope Concentration, are more ambiguous, partly because dispersion parameters require higher sample sizes than do central tendencies. However, some individual cases with more robust signals suggest an increase in Slope Concentration after economic changes. In contrast to the slope coefficient of the mode, the average slope coefficient for the concentration parameter seems to agree across the three technologies, showing in all cases positive values indicative of a lower variance in the Gini coefficients.

### 1.3. Change Point Analysis.

The change-point analysis indicates probabilities of the existence of change points for points in time. The results (*Materials and Methods* and *SI Appendix*) do not show conclusive change points in the development of Gini values. We expected change points to occur in tandem with the introduction of plowing (Europe dt3 = 3700 BCE or 1500 BCE; West Asia dt3 = 3500 BCE). In fact, the probability of identifying a change point in Western Asia and Cyprus is almost the same over the entire period. In Europe, the probability for change points is slightly higher between 4600 and 3300 BCE. Nevertheless, there is no clear correlation in time between traction and housing disparities.

## 2. Discussion

It is a longstanding assumption that the degree of economic inequality, which we define as the accumulation of wealth in certain parts of the society, depends on the production of surpluses—in particular, the development of material productivity (e.g., refs. 22, 31). There is nothing in the GINI data to suggest that land was scarce in the first two millennia of the Neolithic (32), which is why we limit our discussion to labor productivity as a source of possible surplus. We have shown that changes in modes of production can occur without significantly affecting wealth inequality, but lasting wealth inequality needs surplus in order to be realized. We have calculated Gini coefficients for housing disparity. The Gini coefficient measures the deviation of wealth from a hypothetical equal wealth distribution in society. It thus shows and evaluates the existence and extent of societal differences in wealth, in our case, through differences in the sizes of residences.

There are undoubtedly limitations to the possible statements and principal sources of error (30). Our aim is to identify central tendencies in the data. Our worldwide coverage across space and time was limited because the GINI project was based on an opportunistic sampling strategy and rested in considerable part on published datasets. Temporal coverage before and especially after the introduction of plant production also varied considerably across sites (*SI Appendix*). Our statements are limited to inequalities at the site level, i.e., to inequalities referred to as within-group or alpha-inequality (27).

In the following, results are discussed by six regional case studies (Section 1 and *SI Appendix*). The analysis is aggregated at a regional level and therefore no conclusions should be drawn for subregional subsets. We tracked qualitative leaps in productivity development (dt, dt2, dt3) along the time axis. We looked for changes in wealth accumulation that correlated with innovations in mode of production over time. We define our terms as follows. Plant production without animal traction is referred to as horticulture, typically with intensive use of gardens and constrained by the availability of human labor, the energetic limitation on total productivity, and the main defining economic factor (32, 33). The eventual development of irrigation systems and expansive terracing, neither of which is present in our Neolithic data, make it clear that neither the landscape production volume nor the degree of joint cooperation in such systems needs to be low when labor is accumulated over time. The volume of energy per capita is multiplied by the use of animals: if traction enhances crop production, especially seedbed preparation and transport, this is referred to as arable farming. In general, traction results in a lower intensity per area and higher productivity per capita compared to horticulture (34). Herd management means seeking out food resources with animals thus developing new, previously marginal areas of production, whereby there may be a shortage of arable land in society as a whole or between groups. The integration of the three innovations analyzed here, very likely had different practical consequences in different regions (*SI Appendix*) opening up different economic options, some of which may have been additive, leading to a profound systemic change. The latter, in all its consequences, has long been discussed especially for the introduction of traction (e.g., ref. 31). When they occur together, these innovations must be regarded as systemically linked.

To interpret the results of the beta regression (Fig. 3), an increasing Slope Mode value indicates increasing inequalities with an increasing number of sites having higher Gini coefficients. An increasing Slope Concentration indicates a decreased dispersion of the Gini values (i.e., site Gini values are more concentrated around the mode). The sample size is reflected in the posterior ranges: the smaller the sample sizes, the larger the posterior range. Only dt, dt2, or dt3 values whose 50%-HPDI does not include 0 will be discussed here, notwithstanding the fact that evidence supporting any pattern based on this threshold should be interpreted with caution. As mentioned, the beta-regression does not give a clear result here, rather we are in the 90 to 50% HPDI range, which at best allows us to recognize putative trends.

After dt, there is a weak global trend (for all the nine regions combined, the 50% HPDI does not include 0) toward rising and at the same time more concentrated Gini coefficients. Further but similarly weak decreasing dispersions of the coefficients (higher Slope Concentration) are also associated with dt2 and dt3, but there is a tendency for the Gini coefficients to decrease (Slope Mode), meaning that there is lower residential disparity with less variation in inequality.

In E Asia (for a more detailed description of the case study regions, see *SI Appendix*), none of the three innovations resulted in increased Gini values, though plant cultivation makes Ginis more similar across sites, whereas animal domestication makes Ginis less similar across sites (both at low probability). In parts of E Asia, however, dt2 precedes dt chronologically.

W Asia and Cyprus and W and C Europe correspond in Slope Concentration and, for dt as well as dt2, also in Slope Mode, indicating the historical dependency of the West. For dt and dt2, the Gini values are increasingly equalizing in both regions (Slope Concentration), but this trend is no longer observable with dt3. While the change-point analysis for W Asia (*SI Appendix*, Fig. S1) remains undetermined, there is a certain probability of increases in the Gini coefficient in Europe in the time range in which the first traction spread (*SI Appendix*, Fig. S2).

Despite the common history (*SI Appendix*), SE Europe does not follow the W Asian and C and W European pattern. Here too, the Slope Concentration of the Gini coefficients increases with dt, and in line with W and C Europe, the Gini values fall with dt2 (Slope Mode). With dt3, however, the Gini values (Slope Mode) decrease while the dispersion of the Gini values increases.

In N America, too, Gini coefficients increase with dt (Slope Mode). In the Great Plains and in SW North America, the Gini values (Slope Mode) decrease with dt2 and dt3. A clearer decrease in the dispersion of the Gini values (Slope Concentration) can be observed in NE and SE North America. The d2 and d3 transitions have no bearing on the SE and NE N America data, and dt3 can be ignored for all the North American regions.

As the W Asian and European cases show, no substantial difference exists between regions with cores of primary domestication (cf. ref. 35 and those without. The difference is in the time lag between the earliest and the common dt for plant production: the introduction of new elements in the course of a migration shows no time lag, whereas domestication or a gradual takeover takes longer—there are no differences to be expected with regard to dt common.

We already have conceptualized economic innovation as a fundamental expansion of the possibilities for increasing productivity. In general, with dt, both the Slope Mode and the Slope Concentration of Gini values increase. It can only be assumed that the new way of life opened up new room for maneuver, which was initially relatively equal for everyone and at the same time enabled a higher surplus with the possibility for greater wealth inequalities. A further potential increase in productivity (dt2 and dt3), which tends to go hand in hand with greater equality, can be explained by collective field systems, for example.

In the current anthropological, sociological, political, and archaeological discussion about nonstate societies (e.g., refs. 36–38), various leveling mechanisms play a major role. However, attempts to identify such mechanisms archaeologically suffer from a major limitation: so far, mostly the eventual irregular destruction of wealth as potential surplus has been recognized (cf., ref. 2; that is the tip of an unequal distribution of wealth is cut off. The economically balancing effects of feasting remain just as unclear as is the case also of prestigious gifts to the common good. Regular mechanisms that attempt to raise the lower limit of wealth distribution have not been adequately considered—they hardly remain archaeologically visible and adequate proxies have yet to be developed. Thus, we have no quantitative data that assesses the significance of leveling measures.

### 2.1. Melanesian Ethnographic Analogies.

As discussed above, marked economic inequalities did not emerge immediately after the introduction of cultivation, but a considerable time after. This is consistent with expectations from the ethnographic record. The six New Guinea horticulturalist communities investigated are of comparable sizes to those thought to be behind most archaeological sites that date after dt and predate dt3 [mean Gini 0.25, SD = 0.06; (NOfLevels) = 1]. There, economic inequality was minimal under extensive production characterized by first farming (fallow > 10 to 12 y). In these Great-men societies (politics was gendered), leadership was either absent or emerged primarily from a struggle for dominance and prestige, which in turn derived largely from performance in hunting, ritual expertise, and in particular warfare (39–42), none of which appears to have generated, or been accompanied by, economic inequalities. In more intensive horticultural societies (fallow < 10 to 12 y), Big-men eclipsed Great-men, gaining prestige and power from performance in organizing and manipulating conspicuous material displays (40, 42, 43). In contrast to Great-man societies, Big-man societies were characterized by economic inequality: big-men commanded more pigs, shell-wealth, and other materialities than ordinary people. However, these were gift-economies (44, 45) in which a Big-man gained prominence by giving this wealth away not by sequestering it to himself. Wealth flowed through his hands, but very little remained there. As a result, economic inequality did not manifest in ongoing differentials in the possession of stocks of wealth, and because personal conspicuous consumption was minimal, it did not show up as marked size differences in residential units. It is clear that the profit of leveling is dominance and prestige.

## 3. Conclusion

The first 100 Neolithic generations’ development of wealth inequality occurred gradually, along regionally different Neolithic pathways. In the first 2,000 y of the Neolithic, we do not find marked differences in residential disparities in our global sample or in our regional case studies. In fact, we have identified a trend in which innovation tends to distribute wealth more equally in society. Our ethnographic analogies show ways in which comparable societies can deal with wealth inequality. At that stage, the wealth available to society as a whole is likely to have been rather low. Newly brought-in domesticates had to adapt, and the natural conditions had to be adjusted.

The only source of physical power initially available in such societies was human labor (22, 33). The mobilization of this labor force—if we follow our ethnographic analogies—is a crucial economic and political task for Neolithic actors, whether in a bottom–up process or through actors resembling the role of Melanesian big men. All this must have allowed for only a small degree of land development through clearing, well construction, terracing, paving paths, or irrigation. Nevertheless, the period of time for the development of landesque capital (46) cannot be considered as being short.

Where it occurs, the system-change from horticulture to arable farming appears to have eliminated existing residential disparities. Farm sizes were now most likely determined by the number of oxen teams and therefore often of equal size (34): without land shortage, the sizes of farms tend to become equal. North America, the Great Plains and SW, the NE, and SE show rising residential disparities following dt but a similar transition from horticulture to arable farming cannot be monitored since dt2 and dt3 are largely irrelevant in these areas.

Approximation of potential and realized production volumes in these societies is difficult. The fact that potential added value from innovations was not translated immediately into residential disparity indicates to us that it was either not generated (i.e., the fruits of increased productivity were directly consumed as labor savings) or not accumulated. The archaeologically most recognizable indication of egalitarian relations is extensive standardization of property, including house sizes.

Even using criteria as weak as the 50% HPDI and accepting those only for rising inequality, not against it, there is no tendency toward rising wealth inequality following the technological innovations that in the long term caused the most radical systemic changes in prehistoric productivity. On the contrary, these criteria suggest an equalizing effect, but even this effect is not strongly demonstrable at the regional level. We expect significant effects from future smaller-scale regional studies. It will have to be examined whether new technologies were not chosen precisely because of an actual or supposed leveling effect. A new technology, a new economic system may initially have opened up new opportunities for more players than before.

The extent of wealth inequality is undoubtedly linked to productivity: rising productivity, which leads to higher surpluses, is a necessary condition for greater wealth inequality. However, there is nothing to suggest that rising productivity is a sufficient condition for such inequality. In the residential-disparity data examined here, we found no compelling link between three key innovations—plant management, animal management, and animal traction—and rising inequality over the first 2,000 y of the Neolithic. For those two millennia, societies organized and reorganized their economic systems and perhaps increased their productivity without increasing wealth inequality markedly.

## 4. Materials and Methods

### 4.1. Data.

All archaeological data on residential units size were obtained from the GINI project database [(30), for data and software availability see *SI Appendix*]. Gini coefficients were computed at the site level considering only settlements with at least five penecontemporaneous residential units. For each site we computed three variables (dt_plant common_, dt_animal common_, and dt_traction common_) representing the number of years before or after dt, dt2, and dt3 [derived from the fields (Plant Common), (Animal Common), and (Traction Common)]. Site dates were obtained using the midpoint between the fields (BeginDate) and (EndDate). We combined different (Region) fields to obtain nine larger regions for the Beta regression analyses (*SI Appendix*).

### 4.2. Beta Regression.

We compared the distribution of Gini coefficients 2,000 y before (−2,000 < dt_index_ < 0) and after (0 < dt_index_ < 2,000) a particular economic practice has become common, fitting a hierarchical Beta regression model. Beta regression provides two benefits in our case: 1) it is bounded between 0 and 1; 2) it allows the modeling of a concentration parameter in case observed data shows changes in the variance rather than the central tendency (47). More formally, we modeled the distribution of Gini values *g_i,j_* for site *i* in region *j* as follows:

*g_i,j_* ~ Beta(μ_ij_,ϕ_ij_)
  logit(μ_ij_) = α_j_ + β_j_ k_i_
  log(ϕ_ij_) = γ_j_ + η_j_ k_i_,

where μ_ij_ and ϕ_ij_ are the mode and the concentration of a reparameterized Beta distribution, k_i_ is an indicator variable equal to 0 when the site *i* predates the introduction of common availability of a focal technology and equal to 1 otherwise, and parameters α_j_, β_j_,γ_j_, and η_j_ are region-specific regression coefficients. We fitted our data using the nimble R package (48) using the following priors and hyperpriors:

α_j_ ~ normal(μ_α_,σ_α_)
  β_j_ ~ normal(μ_β_,σ_β_)
  γ_j_ ~ normal(μ_γ_,σ_γ_)
  η_j_ ~ normal(μ_η_,σ_η_)
  μ_α_,μ_β_,μ_γ_,μ_η_ ~ normal(0,0.5)
  σ_α_,σ_β_,σ_γ_,σ_η_ ~ exponential(1)

Our primary parameter of interest are the region-specific slopes for the mode (β_j_) and the concentration (η_j_) as well as the panregional average slope defined by μ_β_ and μ_η_. Model fitting was performed for each of the three economic practices separately (dt, dt2, dt3) using four chains, with 200,000 iterations, half discarded for burn-in and with posterior samples collected every 10 steps. We evaluated model convergence by checking the Gelman–Rubin statistic.

### 4.3. Change Point Analyses.

We examined whether the temporal trajectory of the Gini coefficient experienced any notable change in its trajectory by fitting a Bayesian change point model via the *mcp* R package (49). More specifically, we fitted a Gaussian model, with observed distribution of Gini coefficients normally distributed SD σ and a mean μ_i_ defined with the following linear model:

α + β_1_ t_i_, for t_i_ < *z*
  α + β_1_z + β_2_ t_i_, for t_i_ ≥ *z,*

where α is the intercept, β_1_ and β_2_ are slope coefficients before and after the change point *z*, and t_i_ is the date of the site *i*. We use default priors implemented by the *mcp* package for all parameters and fitted our model to two subsets of sites in the Gini database: the “W Asia and Cyprus” and the “Europe” (*SI Appendix*).

## Supplementary Material

Appendix 01 (PDF)

## Data Availability

All scripts and data for replicating the analyses and reproducing main and supplementary figures data have been deposited in tDAR (https://core.tdar.org/project/496853/the-global dynamics-ofinequality-gini-project) (50).
