# Supplementary material for: 100 generations of wealth equality after the Neolithic transitions
Source: Proc Natl Acad Sci U S A. 2025 Apr 14;122(16):e2400697122. doi: 10.1073/pnas.2400697122 (PMC12036991; doi:10.1073/pnas.2400697122)
Supplement: Supplementary file 1 — Appendix 01 (PDF) [file pnas.2400697122.sapp.pdf]

Supplementary Materials

Data, Materials, and Software Availability

All scripts and data for replicating the analyses and reproducing main and supplementary figures are provided in this tDAR Project (https://core.tdar.org/project/496853/the-global-dynamics-of-inequality-gini-project).

Definition of Regions

Aggregated regions are used for Beta-Regression, Box Plots and Scatter Plots. Case studies are marked with an asterisk.

**Aggregated Region [NewRegions]**	**GINI database [Region]**
E Asia*	E Asia
W Asia and Cyprus*	W Asia and Cyprus
SE Europe*	SE Europe
W and C Europe*	W Europe+C Europe
GreatPlains and Southwest NA*	Great Plains+Southwest NA
Northeast and Southeast NA*	Northeast NA+Southeast NA
Southern Mexico and Maya	Southern Mexico+Maya+Central Mexico
Andes	Central Andes+Southern Andes
Africa	S Africa+Horn of Africa+E Africa+W Africa
Global	Contains all regions from above

Aggregated regions used for Change Point analysis:

**New Aggregated Region**	**GINI database**
W Asia and Cyprus	W Asia and Cyprus [NewRegions]
Europe	Europe [Bigregion]

Map (fig.1 of main text)

A map was designed using QGIS version "3.16.12-Hannover" with the "WGS 84 / World Equidistant Cylindrical (EPSG:4087)"-Projection. The data file used is SiteGiniLevel.csv (see SI Data). Dt-common-value isolines were calculated on three local IDW-grids for each partial map.
For the IDW calculation the native QGIS "IDW interpolation" algorithm was used. Interpolation values are retrieved from entries of the "SiteGiniLevel.csv" within the given extent for each region (see 'EXTENT'-Parameter in the "idw-parameters.md"-file) and saved in regional GeoPackages. Interpolation attribute for all regions is "Plant.cultivation...common". The parameters for each region are listed in the "idw-parameters.md"-file. Iso-lines are extracted using the native QGIS Contour algorithm on each IDW grid. The parameters for each region are listed in the "idw-parameters.md"-file. For clarity, only selected and shortened isolines are displayed on the map. For displaying "before dt-common" and "after dt-common", two different styles were used, which are provided in data repository (before_dt_common.qml and after_dt_common.qml).
Proj4-Definition for map projection: "+proj=eqc +lat_ts=0 +lat_0=0 +lon_0=0 +x_0=0 +y_0=0 +datum=WGS84 +units=m +no_defs".
The Base maps were provided by naturalearthdata.com with the datasets "ne_50m_land", "ne_10m_lakes", "ne_50m_admin_0_boundary_lines_land", "ne_50m_admin_0_boundary_lines_disputed_areas", "ne_50m_admin_1_states_provinces_lines".

Change Point analysis

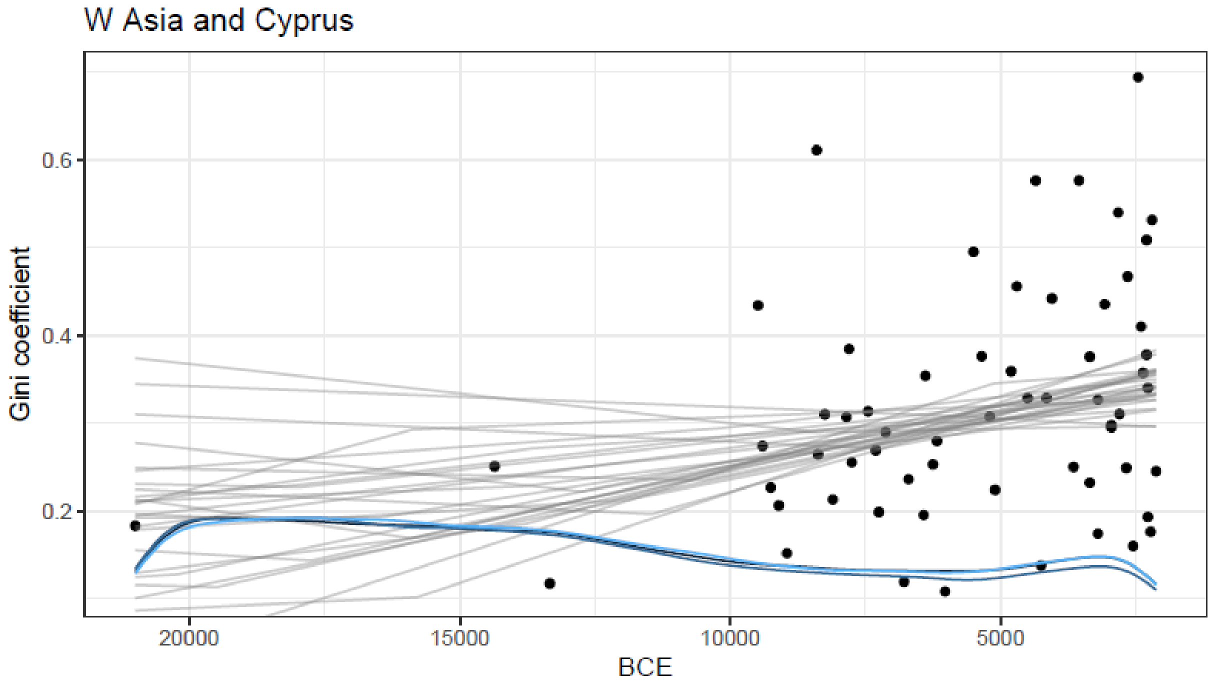

Fig. S1: Change point analysis for Western Asia and Cyprus. For data and method see main text (4.3. Materials and methods), for the regions see above in the SI.

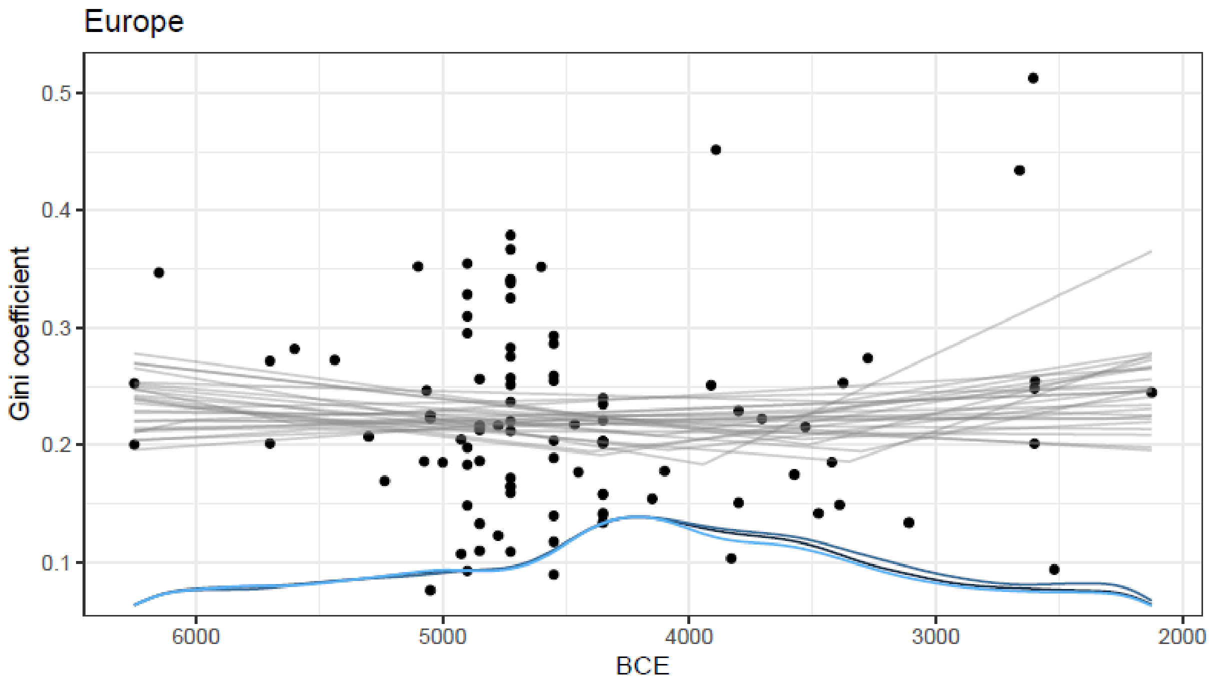

Fig. S2: Change point analysis for Western Europe. For data and method see main text (4.3. Materials and methods), for the regions see above in the SI.

Case Studies (references see below)

Here, the six case studies with sufficient data coverage are discussed in detail. For the other three regions please go to the original data.

E Asia

The East Asian dataset consists of 14 Neolithic sites from China, 86 Bronze Age (Mumum period) sites from Korea, 30 hunter-gatherers (Jomon period) and 30 early farming to Bronze Age (Yayoi period) sites from Japan. In N China, dt2 occurs as early as 6500 BCE, followed by earliest plant management at 6200 BCE and dt3 at 500 CE. In Korea, cultivation of millet started around 2500 BCE during the Chulmun period, but its impact on the subsistence economy became prominent only from ca. 1500 BCE, when rice cultivation was introduced. In Japan, the earliest evidence of rice and millet cultivation dates back to the start of the 1st millennium BCE. Our analyses show no discernible change in residential disparity before and after dt and dt2 (fig.2 and fig.3), with only weak evidence (i.e. 50% but not the 90% HPDI not including 0) suggesting a shift towards a reduced variability in the Gini coefficient after dt and an increased variability after dt2. Given the limited representativeness of the Chinese data, the overall pattern fundamentally reflects the samples from Korea and Japan, with samples before farming entirely represented by a few Jomon sites within the 2,000-year envelope. In the case of Japan, it is worth noting that the prominence and impact of farming were comparatively limited during the early stages of the Yayoi period (Stevens et. al. 2022), and while there is evidence of large burials requiring the coordination and mobilization of labor forces (Mizoguchi 2013), these are comparatively limited in size and rather frequent compared to later, substantially larger burial tumuli observed in the Kofun period. Our sample from Korea is chronologically limited to the Middle Mumun period, hence we were not able to determine whether the shift from a collective and egalitarian ideology of the Early Mumum to the emergence and consolidation of elites in the Late Mumun period suggested from changes in burial practices (Kim 2014) were reflected also in patterns of residential disparity.

W Asia and Cyprus

This relatively large subset of data covers the entire transition sequence from hunter-gatherers to farmers using traction on arable fields. Of the 93 sites, 64 are older than 1500 BCE (2000 years after dt3, fig. in SI). The two sites with the highest Gini coefficients (>0.7, Kültepe, Ur) were Bronze Age urban centers (Lawrence et al. 2021). Abu Salem also has a notably high Gini value (>0.6), which may derive from all or some of the buildings functioning as storage facilities rather than residences (Gopher et al. 1998). In the Fertile Crescent there are two levels of settlements until the Chalcolithic, those of 'normal' size and anomalously large ones. Our data set does not include any of the anomalous large settlements, but there are no indications that the two differ significantly in terms of the variability and range of residence size or floor plan. This means [NOflevels] is no more than 2. In SW Asia, domestication is relatively slow, with great diversity in subsistence practices and, at times, a return to hunter-gatherer lifestyles. However, within the period dt 2000 to dt 4000 (6000 BCE to 4000 BCE), numerous economic and social changes occurred (Basri and Lawrence 2020) that are generally associated with the Chalcolithic (dt3 after 3500 BCE). Increasing signs of urbanization and the emergence of archaic states (Feinman and Marcus 1998) coincide with innovative technologies such as new metallurgical techniques, intensified wool production, oil production, and crop production

which also includes ploughing. V. Gordon Childe's urban revolution, A. Sherratt's secondary products revolution as well as a metallurgical revolution (Levy and Garrett 2007) are linked to this transition and shift in technologies. In W Asia, one of the global core areas of the domestication of animals and so-called founder crops (common in the Pre-Pottery B sites c. 8500 BCE, (Zohary et al. 2012)), Gini coefficients are not rising after dt, dt2 and dt3 (fig. 2). Changes in Slope Mode (fig. 3) are not visible, but changes in Slope Concentration indicate, when applying the 50% criterion, a decrease of dispersion of the Gini coefficients after dt and dt2. No effects of dt3 are visible. In general, the data set can be regarded as representative: This applies both with regard to the settlement hierarchy as represented here (with the exception mentioned above) and with regard to the first or general appearance of the innovations dt, dt2, dt3.

SE Europe

The GINI data spans the south-east European mountains and the neighbouring lowland areas from the Balkan Peninsula. While there is no data before dt earliest, there is reliable coverage after dt and dt2, dt3 is clustering around dt3=1000 (fig. in SI). In these regions, settlement hierarchies are not known. The majority of the Balkan sites are stratigraphically multi-layered sites, yet all sites before 3900 BCE have [NOfLevels]=1. While little is known about the settlement hierarchies in these regions, there exists a strong research bias focused on tells, but we assume that smaller settlements existed between the larger tell sites. Given the restricted nature of tells, areas for settlement were likely scarce (Kerig et al. 2023; Porčić 2019) which may have led to competition for building space. The composition of the cultivated plants, as well as other characteristics of the culture, clearly show the origin of the southern European Neolithic from western Asia. However, both developed apart over time. The introduction of W Asian founder-crops, such as einkorn, emmer, lentil, into SE Europe (c. 6500 BCE) has no effects on Slope Mode but it does for Slope Concentration (fig. 3). In this region, the animal management of dt2 refers to sheep/goats, which can exploit marginal areas. When the 50% HPDI criterion is used, dt2 and dt3 are followed by lower Slope Mode values, while Slope Concentration is higher after dt3. With dt2 at 5500 BCE (earliest c. 7000 BCE (Price 2000)), and dt3 (here most conservatively set to 1500 BCE, but see (Halstead and Isaakidou 2013)) Slope Mode seems to be decreasing, while after dt3 Slope Concentration is rising. In fact, dt3 is heavily biased by a 2000-year gap in the data immediately before Knossos (circa 1600 BCE) with its extraordinarily high Gini coefficient of 0.86.

W and C Europe

The GINI dataset comprises 28 Western and Central European Neolithic sites from a relatively small but contiguous geographical area, with a focus on Switzerland and neighbouring parts of E France and S and W Germany. Hunter-gatherer sites are not available in the region, which makes it impossible to draw conclusions about the earliest effects of Neolithization (dt occurs at 5400 BCE). Nonetheless, the GINI data represents the variation in settlements and settlement systems in the region. Early Neolithic sites are all located on extremely fertile loess soils and early domestic spaces were much larger than later ones (Coudart 1998; Luley 1990). The later development of house construction and settlement layout is well documented in wet-preserved sites: from c. 3500 BCE onwards settlement plans, at least in the circum-Alpine wet preserved sites ('pile dwellings'), show streets which allow the use of travois or wheeled transport (Hafner et al. 2016). Here, too, the data set is small but thoroughly representative of the current state of knowledge. However, this does not change the problematic fact that hardly any settlement activities from the hinterland of the pile dwellings are known. Although larger and smaller settlements coexist in the Early Neolithic, a settlement hierarchy

is not recognizable in either production nor consumption (Hafner et al. 2016), which is why we coded [NOflevels] = 1, as for the entire Neolithic of the region. While the houses of the Early Neolithic probably lasted a generation, the houses of the pile-dwelling settlements often seem to have been in use for only a few years. New evidence indicates that the earliest ploughing began around the 5th millennium (van Willigen 2024) while dt3 is much later (3700 BCE). During the Neolithic an expansion in Neolithic settlement activities occurred from fertile loess-derived soils in earlier periods to less productive marginal areas including wetlands in later periods (Lechterbeck and Kerig 2024). This expansion is accompanied by a general reduction in the size of all buildings, from longhouses to small houses. In the Earliest Neolithic, the W Asian founder-crop package was introduced to the woodlands of temperate continental Europe from SE Europe (Shennan 2018). Evidence from before dt and dt2 is missing (fig. in SI). Most Gini coefficients after dt, dt2 and dt3 remain low, around 0.2, indicating less residential disparity. Following dt is a rising Slope Concentration (fig. 3), the same is true for dt2 while changes in Slope Mode following dt are not visible, dt2 and dt3 are followed by lowering the Slope Mode values.

NE and SE Northern America

Of 105 sites 36 are from the NE, all with positive dt-dates (1150 to 1644 CE), 69 are from the SE (1 to 1768 CE) of which only 7 have negative dt-values (1 to 750 BCE). The NE dataset was compiled based on published and grey literature where complete or near-complete site-level settlement data were available and the SE dataset was assembled following (Steere 2017; Betzenhauser 2018). After dt, inequality increases, visible both in the box plot (fig. 2) and, when applying the 50% criterion, in the distribution of the Slope Mode (fig. 3). In fact, the contrast between the pre- and post-common agricultural threshold is more pronounced here than in any of the other regions (fig. 2). The Slope Concentration does not change after dt but rises after dt2. While the dataset indicates a date of 1600 CE for dt2 transition in North America, the differential impacts of European colonialism east of the Mississippi River resulted in dt2 and dt3 occurring much later in the context of colonial encroachment and entrenchment and are not related to the sample analyzed in this study which derive from Indigenous social contexts. In the NE multi-family longhouses dominated residential settlement patterns without settlement hierarchy (NOfLevels=1) and Gini coefficients never rose above 0.3. SE North America possessed a more differentiated settlement system, particularly during the later prehistoric Mississippian period (where NOfLevels reaches 4) but without developing many high or differentiated Gini coefficients. Where we do see a slight increase in Gini coefficients — in the American Bottom region of SE NA — it is in the ca. 1100-1250 CE period (e.g., Cahokia, Kincaid) (Emerson and Pauketat 1997). Here nascent elites may have utilized surpluses afforded by climatic conditions and ideologically-charged practices to integrate resident and rural populations in sets of unequal relations (Pauketat et al. 2023). However, a general decline in inequality after this brief, locally-specific increase suggests that social institutions shared by societies in each region (Holland-Lulewicz et al. 2022) served to restrict the accumulation of wealth in any one sector of society.

Great Plains and SW North America

This sample is comparatively large (total n=122; Great Plains: 52 sites; Southwest: 70 sites), without significant time gaps. The southwestern sites come largely from the northern reaches of the US SW, including the subregion “Upland SW” (25 sites) and its “Northern Periphery” (Fremont) (29 sites). Within the Great Plains the best-represented subregion is Wyoming (33 sites). Maize is the main cultigen in both regions, although the date for its arrival and degree of importance varied greatly. In the southern SW, maize arrived from Mesoamerica somewhat before 2000 BCE (Vint 2015); in the

Great Plains, maize arrived first to its NE margins ~900 CE (Bamforth 2021), ultimately from the SW, before spreading more widely. The animals considered in the dt2 threshold differed however. For the SW we coded turkey (not dog) as the first domesticate, becoming commonly used for food in some subregions ~1050 CE (Lipe et al. 2016); for the Great Plains we coded horse (not dog) as the first domesticate, becoming common ~1600 CE (Taylor et al 2023). Since there was no significant use of traction at any of the sites in this sample, dt3 is irrelevant. Before and after dt and dt2 slope concentrations for Gini coefficients remain stable (fig. 3). While changes in Slope Concentration do not depart from the 0-line, 50%-Slope Mode shows an increase in inequality following dt but a decrease after dt2 (fig. 3). In other words, the pooled sample for these regions indicates low-probability increases in wealth inequality with agriculture but also low-probability decreases in wealth inequality with animal domestication. The sample (fig. 2) however obscures dynamics known from more detailed analysis at finer temporal and spatial scales using a precursor to the SW dataset analyzed here. Gini coefficients increased rapidly in the Chaco area in the mid-800s CE, with higher (but decreasing) levels of inequality persisting there and in the northern SW more broadly until the mid-1100s CE, when they rapidly declined (Ellyson et al. 2019). Analyses of low-frequency trends in summer temperatures suggest generally increasing aridity throughout the SW after ~1100 CE (Gillreath-Brown et al. 2024). The maize dry-farming niche went through marked constriction in the mid-1100s CE, followed by poor conditions through much of the 1200s CE. The generally low post-Chaco (post-1140 CE) Gini coefficients likely reflect diminished productive capacity at least as much as changed socio-political patterns of surplus distribution (Kohler et al. 2023; Kohler et al. 2025).

References

P. Basri, D. Lawrence, Wealth inequality in the ancient Near East: A preliminary assessment using Gini coefficients and household size. *Camb. Archaeol. J.* **30**, 689–704 (2020).

D. B. Bamforth, The Archaeology of the North American Great Plains (Cambridge University Press, 2021).

A. Betzenhauser, Exploring Measures of Inequality in the Mississippian Heartland. *In* Ten Thousand Years of Inequality: The Archaeology of Wealth Differences, T. A. Kohler, M. E. Smith, Eds. (University of Arizona Press, 2018). pp. 180–200.

A. Coudart, Architecture et société néolithique: l'unité et la variance de la maison danubienne (1998).

L. J. Ellyson, T. A. Kohler, C. M. Cameron, How far from Chaco to Orayvi? Quantifying inequality among Pueblo households. *Journal of Anthropological Archaeology* **55**, 101073 (2019).

T. E. Emerson, T. R. Pauketat, Eds., *Cahokia: Domination and Ideology in the Mississippian World* (University of Nebraska Press, 1997).

G. M. Feinman, J. Marcus, *Archaic States* (School of American Research Press, 1998).

A. Gillreath-Brown *et al.,* A Low-Frequency Summer Temperature Reconstruction for the United States Southwest, 3000 BC – AD 2000. *The Holocene (on-line first)* (2024) https:/doi.org/10.1177/09596836231219482.

A. Gopher *et al.*, A Pre-Pottery Neolithic B Camp in the Central Negev Highlands, Israel. *Bull. Am. Schools Orient. Res.* **312**, 1–20 (1998).

A. Hafner *et al.*, "Ufer- und Moorsiedlungen. Chronologie, kulturelle Vielfalt und Siedlungsformen" in *4000 Jahre Pfahlbauten*, Archäologisches Landesamt Baden-Württemberg, Landesamt für Denkmalpflege Baden-Württemberg, Eds. (Thorbecke, 2016), pp. 60-64.

P. Halstead, V. Isaakidou, "Early stock-keeping in Greece" in *The Origins and Spread of Stock-Keeping in the Near East and Europe*, S. Colledge, J. Conolly, K. Dobney, S. Shennan, Eds. (Left Coast Press, 2013), pp. 129–144.

J. Holland-Lulewicz *et al.,* Keystone Institutions of Democratic Governance Across Indigenous North America. *Frontiers in Political Science* **4** (2022).

T. Kerig *et al.*, "An archaeological perspective on social structure, connectivity and the measurements of social inequality." in: *Connectivity matters! Social, Environmental and Cultural Connectivity in Past Societies*, J. Müller, Ed. (Sidestone press, 2023).

J. Kim, From Labour Control to Surplus Appropriation: Landscape Changes in the Neolithization of Southwestern Korea. *Journal of World Prehistory* (2014). 263–275. https://doi.org/10.1007/s10963-014-9076-y

T. A. Kohler *et al.,* Wealth inequality in the prehispanic northern US Southwest: from Malthus to Tyche. *Phil. Trans. R. Soc.* B37820220298. (2023) https://doi.org/10.1098/rstb.2022.0298

T. A. Kohler, *et al.*, Economic inequality is fueled by population scale, land-limited production and settlement hierarchies across the archaeological record . *Proc. Natl. Acad. Sci. U. S. A.* (2025, this issue)

D. Lawrence, *et al.,* Climate change and early urbanism in Southwest Asia: A review. https://wires.onlinelibrary.wiley.com/doi/full/10.1002/wcc.741 (2021)

J. Lechterbeck, T. Kerig, Inventions, innovations and the origins of spelt wheat. *Veg. Hist. Archaeobot.* (2024) https:/doi.org/10.1007/s00334-023-00978-2.

T. E. Levy, K. Garrett, *Journey to the Copper Age: archaeology in the Holy Land* (San Diego Museum of Man, 2007).

W. D. Lipe *et al*., Cultural and Genetic Contexts for Early Turkey Domestication in the Northern Southwest. *American Antiquity* 81(1) 2016, 97-113.

H. Luley, *Urgeschichtlicher Hausbau in Mitteleuropa : Grundlagenforschungen, Umweltbedingungen und bautechnische Rekonstruktionen* (Habelt, 1990).

K. Mizoguchi, *The Archaeology of Japan: From the Earliest Rice Farming Villages to the Rise of the State*. (Cambridge University Press 2013).

T. R. Pauketat *et al.*, Cahokia as Urban Anomaly. *Journal of Urban Archaeology* **7**, 253–274 (2023).

M. Porčić, Evaluating social complexity and inequality in the Balkans between 6500 and 4200 BC. *J. Archaeol. Res.* **27**, 335–390 (2019).

T. D. Price, Ed., *Europe's First Farmers* (Cambridge University Press, 2000).

S. Shennan, *The First Farmers of Europe: An Evolutionary Perspective* (Cambridge UniversityPress, 2018).

B. A. Steere, *The Archaeology of Houses and Households in the Native Southeast* (University of Alabama Press, 2017).

Stevens, C. J., Crema, E. R., & Shoda, S. The importance of wild resources as a reflection of the resilience and changing nature of early agricultural systems in East Asia and Europe. *Frontiers in Ecology and Evolution* https://www.frontiersin.org/articles/10.3389/fevo.2022.1017909. (2022)

W. T. T. Taylor *et al.,* Early dispersal of domestic horses into the Great Plains and northern Rockies. *Science* **379**,1316-1323(2023).DOI:10.1126/science.adc9691

J. M. Vint, Las Capas, AZ AA:12:111 (ASM), Introduced: Background, Chronology, and Research Orientation. *In* J. M. Vint ed. *Implements of Change: Tools, Subsistence, and the Built Environment of Las Capas, an Early Agricultural Irrigation Community in Southern Arizona*. Anthropological Papers No. 51 (Archaeology Southwest, 2015) pp. 1–32

S. van Willigen *et al.,* New evidence for prehistoric ploughing in Europe. *Humanit Soc Sci Commun* **11**, 372 (2024). https://doi.org/10.1057/s41599-024-02837-5

D. Zohary *et al.*, *Domestication of Plants in the Old World: The Origin and Spread of Domesticated Plants in Southwest Asia, Europe, and the Mediterranean Basin* (OUP Oxford, 2012).

Scatter plots

For R script and data (SiteGiniLevel.csv) see above Supplementary Materials “Data”.

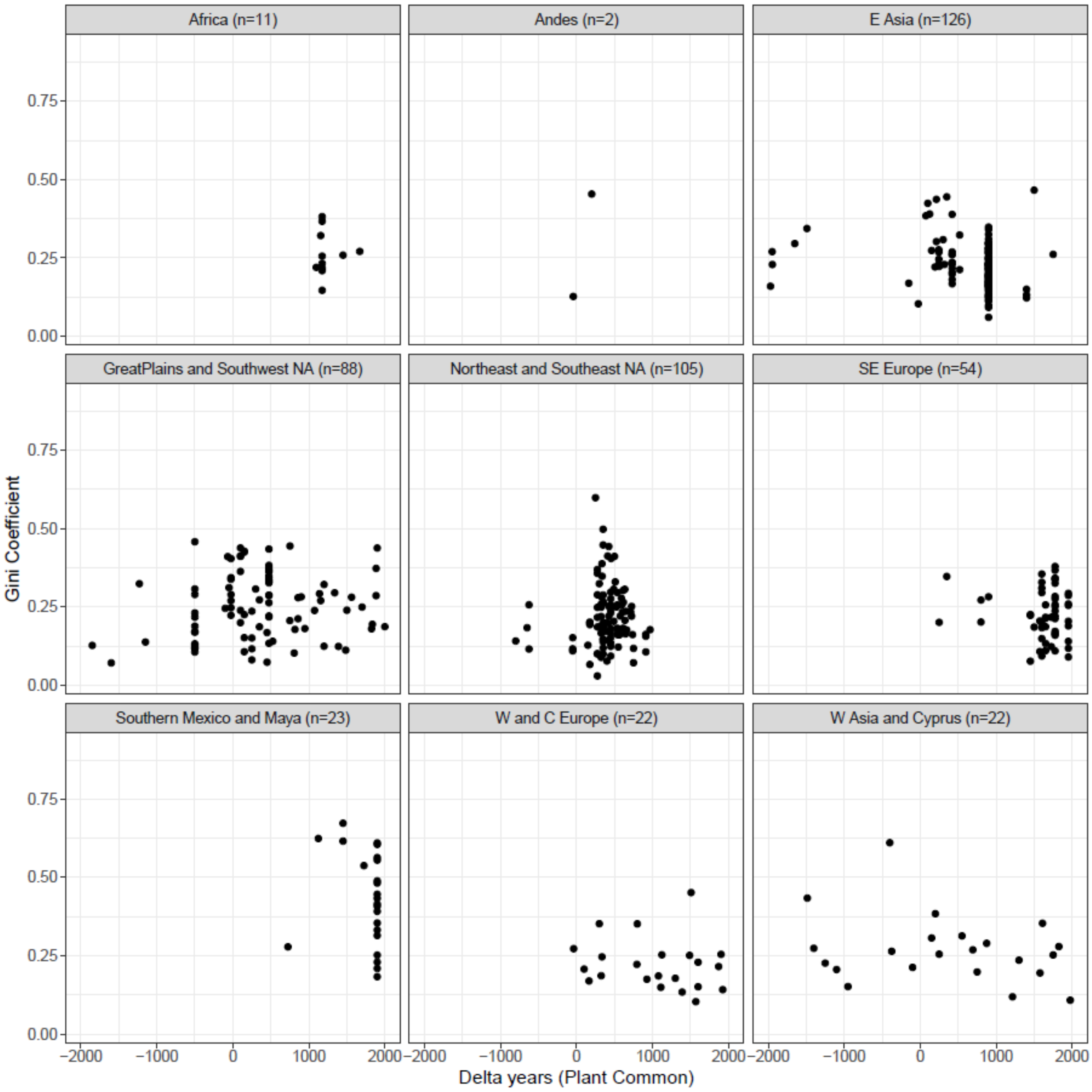

Fig. S3: Effects of the introduction of crops (delta year 0 marks first point in time when domesticated plants were common) on the disparities in residential size (measured in Gini coefficient on site level).

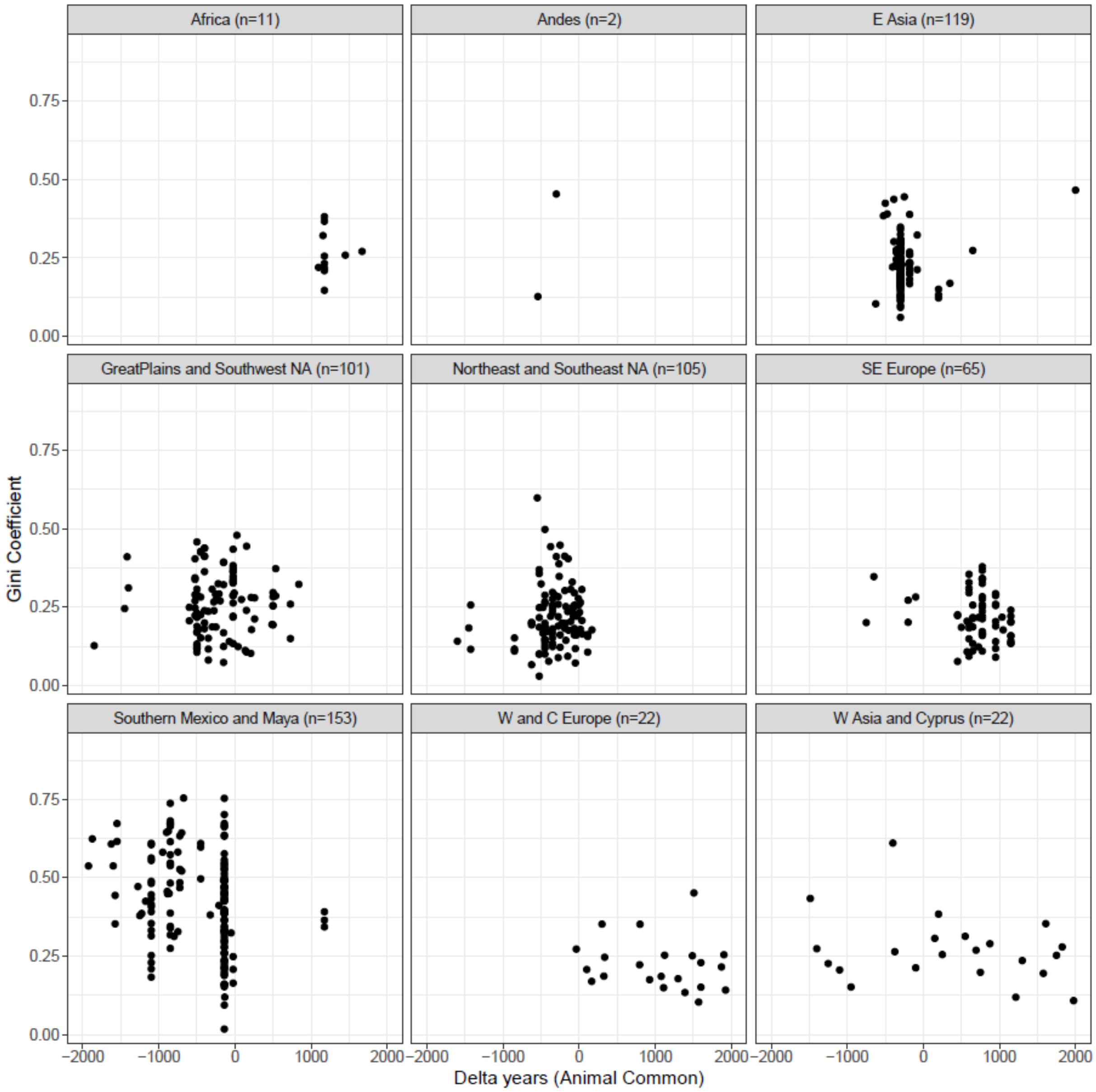

Fig. S4: Effects of the introduction of husbandry (delta year 0 marks first point in time when domesticated animals were common) on the disparities in residential size (measured in Gini coefficient on site level).

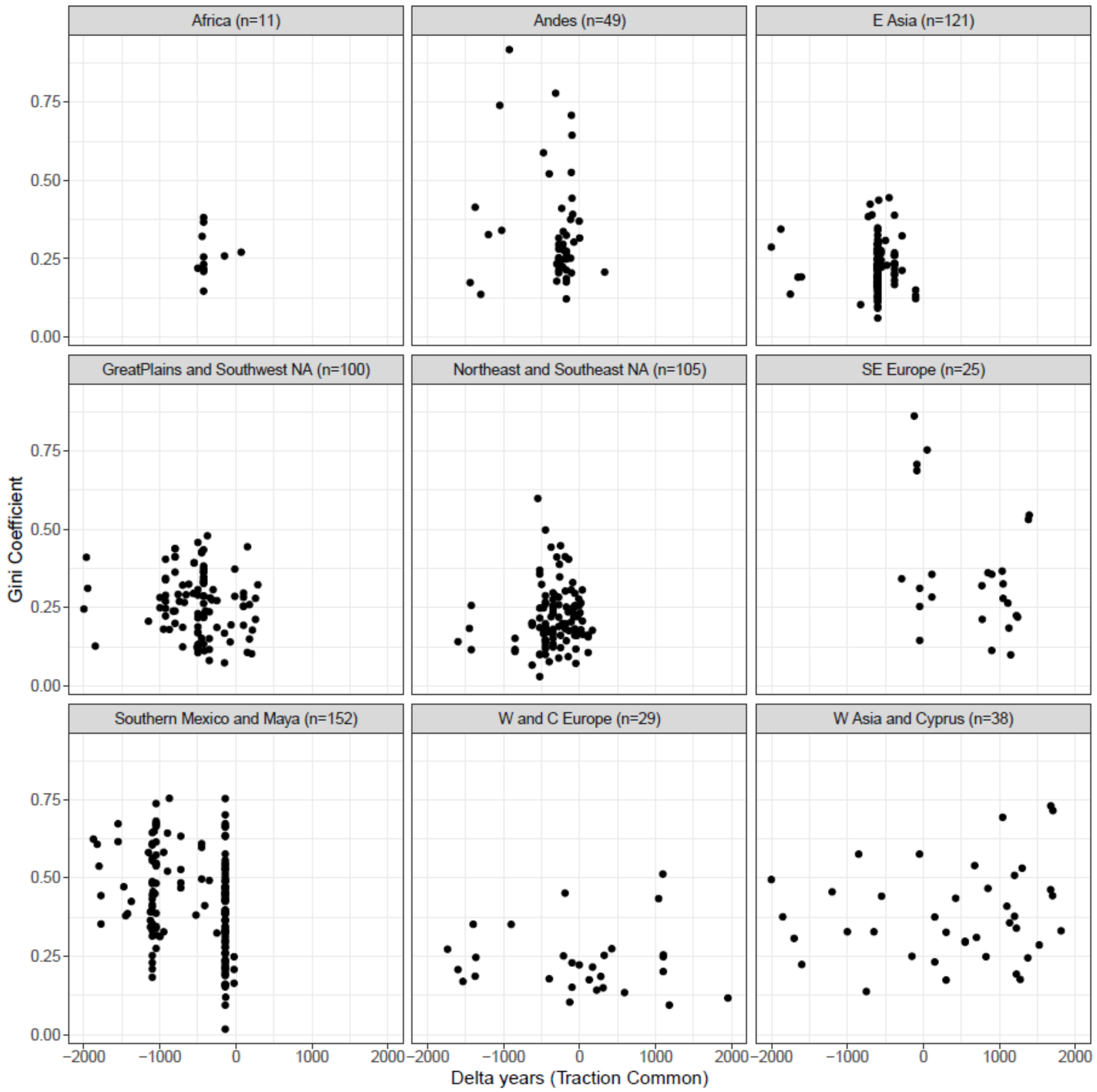

Fig. S5: Effects of the introduction of traction (delta year 0 marks first point in time when animal traction was common) on the disparities in residential size (measured in Gini coefficient on site level).
